# Topic: “Color Image Processing: Models and Methods (CIP: MM)”

**DOI:** 10.3390/e28040383

**Published:** 2026-03-31

**Authors:** Giuliana Ramella, Isabella Torcicollo

**Affiliations:** Institute for the Applications of Calculus “M. Picone”, National Research Council (CNR), Via P. Castellino 111, 80131 Naples, Italy; isabella.torcicollo@cnr.it

Color information plays a crucial role in digital image processing: it is a robust descriptor that can improve data compression and support scene understanding for both humans and automatic vision systems. Color Image Processing (CIP) has become a highly active research area with applications spanning biomedicine, cultural heritage, remote sensing, defense, and security, among others.

This Topic was conceived to provide an overview of the state of the art and stimulate present and future directions, explicitly bridging two aspects often treated separately: the mathematical modeling and computational design of methods. This Topic is hosted across four participating journals (*Applied Sciences*, *Entropy*, *Journal of Imaging*, *Optics*), reflecting the interdisciplinary nature of CIP research.

The Topic brings together 14 peer-reviewed open access papers published between 2022 and 2025, authored by researchers from a wide range of countries. Overall, the contributions highlight how advances in CIP emerge from continuous interplay between model-based formulations, optimization, and learning-based architectures, with strong ties to real-world constraints and application-driven evaluation. Taken together, the papers in this Topic confirm that CIP remains a fertile domain in which theory, computation, and data-driven methods mutually reinforce one another.

The published papers reflect the breadth of CIP models and methods and span several interconnected directions:

- Imaging systems, sensing, and multimodal perception, including hyperspectral imaging design/optimization and assistive perception systems.
- Segmentation, thresholding, and detection in complex scenes, covering optimization-driven color segmentation and cross-modal (RGB-thermal) object detection.
- Restoration and enhancement of challenging imagery, addressing dehazing, nighttime imaging/stitching, and quality-oriented fusion pipelines.
- Color transfer, representation, and multivariate operators, including example-based color transfer, order-statistics morphology for color data, and hue-driven pixel manipulations in graphics workflows.
- Learning-based reconstruction and detail recovery, including super-resolution and reference-guided multi-exposure fusion.
- Biomedical and medical imaging applications, from retinal fundus multi-label classification to pseudo-coloring of grayscale nuclear imaging for improved interpretability.
- Color analysis beyond pure vision tasks, such as quantitative chromaticity analysis in cultural and landscape contexts.

We would like to thank all authors for their valuable contributions and all reviewers for their careful evaluations and constructive feedback. We are also grateful to the Topic Editors and the Editorial Offices of the participating MDPI journals for their support in managing and coordinating this cross-journal Topic initiative.

